# Roles of N6‐methyladenosine epitranscriptome in non‐alcoholic fatty liver disease and hepatocellular carcinoma

**DOI:** 10.1002/SMMD.20230008

**Published:** 2023-06-02

**Authors:** Yuyan Chen, Zhengyi Zhu, Lu Zhang, Jinglin Wang, Haozhen Ren

**Affiliations:** ^1^ Department of Hepatobiliary Surgery Nanjing Drum Tower Hospital Affiliated Hospital of Medical School Nanjing University Nanjing China; ^2^ Department of Hepatobiliary Surgery Nanjing Drum Tower Hospital Clinical College of Xuzhou Medical University Nanjing China

**Keywords:** epitranscriptomics, hepatocellular carcinoma, N6‐methyladenosine modifications, non‐alcoholic fatty liver disease

## Abstract

Non‐alcoholic fatty liver disease (NAFLD) is a typical chronic liver disease connected to a high risk of developing hepatocellular carcinoma (HCC). The development of NAFLD and HCC has been associated with changes in epigenetics, such as histone modifications and micro RNA (miRNA)‐mediated processes. Recently, in the realm of epitranscriptomics, RNA alterations have become important regulators. N6‐methyladenosine (m6A) is the most common and crucial alteration for controlling mRNA stability, splicing, and translation. It is particularly important for controlling liver disease progression and hepatic function. This review aims to conclude recent research on the functions of m6A epitranscriptome in the molecular mechanisms behind NAFLD and HCC development, with special attention to the effects of m6A alteration on how HCC develops and its possible roles in the progression of NAFLD to HCC. Additionally, the review discusses the possible effects of m6A alteration on the treatment and diagnostic of NAFLD and HCC. It is crucial to remember that m6A modification is a reversible action controlled via the coordinated functions of the proteins that write and delete, enabling quick adaptability to environmental changes. The review also discusses m6A‐binding proteins' function in mRNA alternative splicing, translation, and degradation and their ability to modulate mRNA stability and processing. Understanding RNA modification regulation and its part in the emergence of HCC and NAFLD may provide new avenues for diagnosing and treating these diseases.

## Graphical abstract

The m6A modification serves as a vital role in the progression of non‐alcoholic fatty liver and hepatocellular carcinoma.

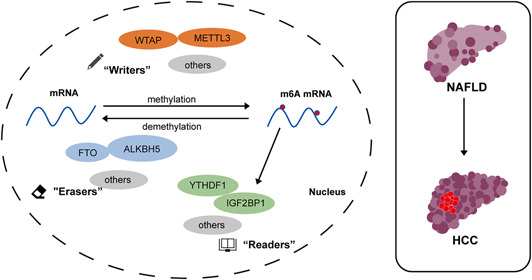

**Key points**

- N6‐methyladenosine (M6A) is the most common and crucial alteration for controlling mRNA stability, splicing, and translation in non‐alcoholic fatty liver disease (NAFLD) and hepatocellular carcinoma (HCC).
- M6A methylation modification is crucial for the lipid metabolism, cell apoptosis, and autophagy in NAFLD.
- Increased levels of m6A methylation correlate positively with the degree of malignancy in HCC patients, which also involved in the biological processes of HCC proliferation and metastasis.

## 1 INTRODUCTION

**FIGURE 1:**
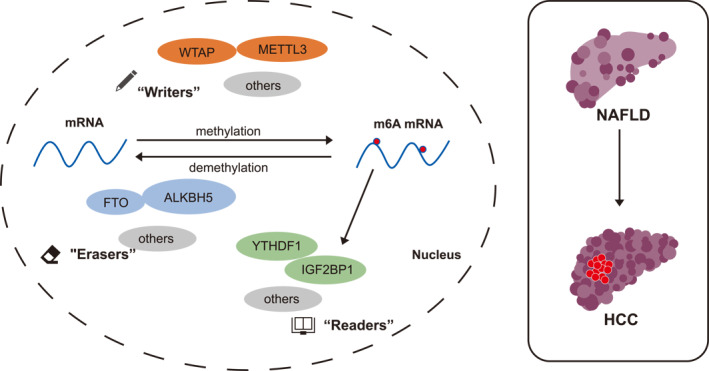
The m6A modification serves as a vital role in the progression of NAFLD and HCC. HCC, hepatocellular carcinoma; m6A, N6‐methyladenosine; NAFLD, non‐alcoholic fatty liver disease.

Hepatocellular carcinoma (HCC) is the leading cause of cancer‐related mortality worldwide, accounting for 80%–90% of all primary liver cancer cases.^1^ The etiology of HCC is multi‐faceted, of which the most common cause is chronic liver inflammation caused by hepatitis B virus (HBV) and so on, followed by alcoholic as well as non‐alcoholic fatty liver disease (NAFLD).^2^, ^3^, ^4^ Recent epidemiological studies have attributed the increasing frequency of HCC to NAFLD, due to the prevalence of obesity and the metabolic syndrome worldwide led by caloric excess and sedentary lifestyle, and the development of effective vaccines for HBV‐ and potent anti Hepatitis C virus‐ drugs.^5^ Despite the well‐defined risk factors, the underlying molecular pathways are unclear in the establishment of HCC and NAFLD, and their relationships remain ambiguous (Figure 1).

Mounting evidence has shown that typical epigenetic changes, including DNA methylation, histone modification, and micro RNA (miRNA)‐mediated processes, are critically associated with the development of NAFLD and HCC.^6^ Recently, significant advances in the regulation of gene expression by RNA modification have given rise to the new science of epitranscriptomics.^7^ Since the advent of high‐throughput sequencing technology, over 170 distinct post‐transcriptional RNA modifications have been noticed.^8^ In response to the abundance of RNA changes, the term “Epitranscriptome” was first coined in 2012,^9^ which is similar to the idea that DNA or histone changes can change the way genes work. Most RNA modifications are found in transfer RNA and ribosomal RNA, but only a few have been found in mRNA. These include N6‐methyladenosine (m6A), N1‐methyladenosine (m1A), 5‐methylcytosine (m5C).^10^ M6A, the most prevalent type of eukaryotic internal messenger RNA modification, is crucial for controlling messenger RNA (mRNA) stability, splicing, and translation as well as for controlling the function of the liver and the emergence of liver disorders, particularly NAFLD and HCC.^6^ Although the presence of m6A in polyadenylated mRNA was initially found in the 1970s, the impact of these changes on gene expression regulation has only recently begun to be investigated.^11^

The most abundant modification on mRNA is the methylation of adenylate N6 (m6A), which is involved in all aspects of the mRNA life cycle. Studies have shown that the most important known function of m6A is to regulate the stability of mRNA: The mRNA modified by m6A in the cytoplasm can be recognized by YTHDF2 to enrich it into the Processing body (P‐body), thus accelerating the degradation of mRNA. In addition, m6A modification can also change the secondary structure of RNA and regulate target recognition of microRNA to regulate the stability of mRNA. In the nucleus, m6A modification can regulate RNA splicing and nucleation process, thus regulating gene expression. m6A may also interact with DNA methylation. Studies have shown that FTO Alpha Ketoglutarate Dependent Dioxygenase (FTO) can inhibit the Wnt/β‐catenin signaling pathway, thus affecting the proliferation and differentiation of porcine intraminogenic preadipocytes.^12^ FTO can also down‐regulate the m6A level of human hepatoma cells HepG2, reduce mitochondrial content, and increase triglyceride deposition, which provides a new idea for the early prevention of NAFLD.^13^ In addition, the regulatory protein of m6A methylation has many potential targets for HCC therapy. Studies have shown that the methylation level of m6A is closely related to the prognosis of HCC, and YTHDF2, YTHDF1, METTL3, and KIAA1429 may be potential predictors and therapeutic targets of HCC.^14^

**FIGURE 2:**
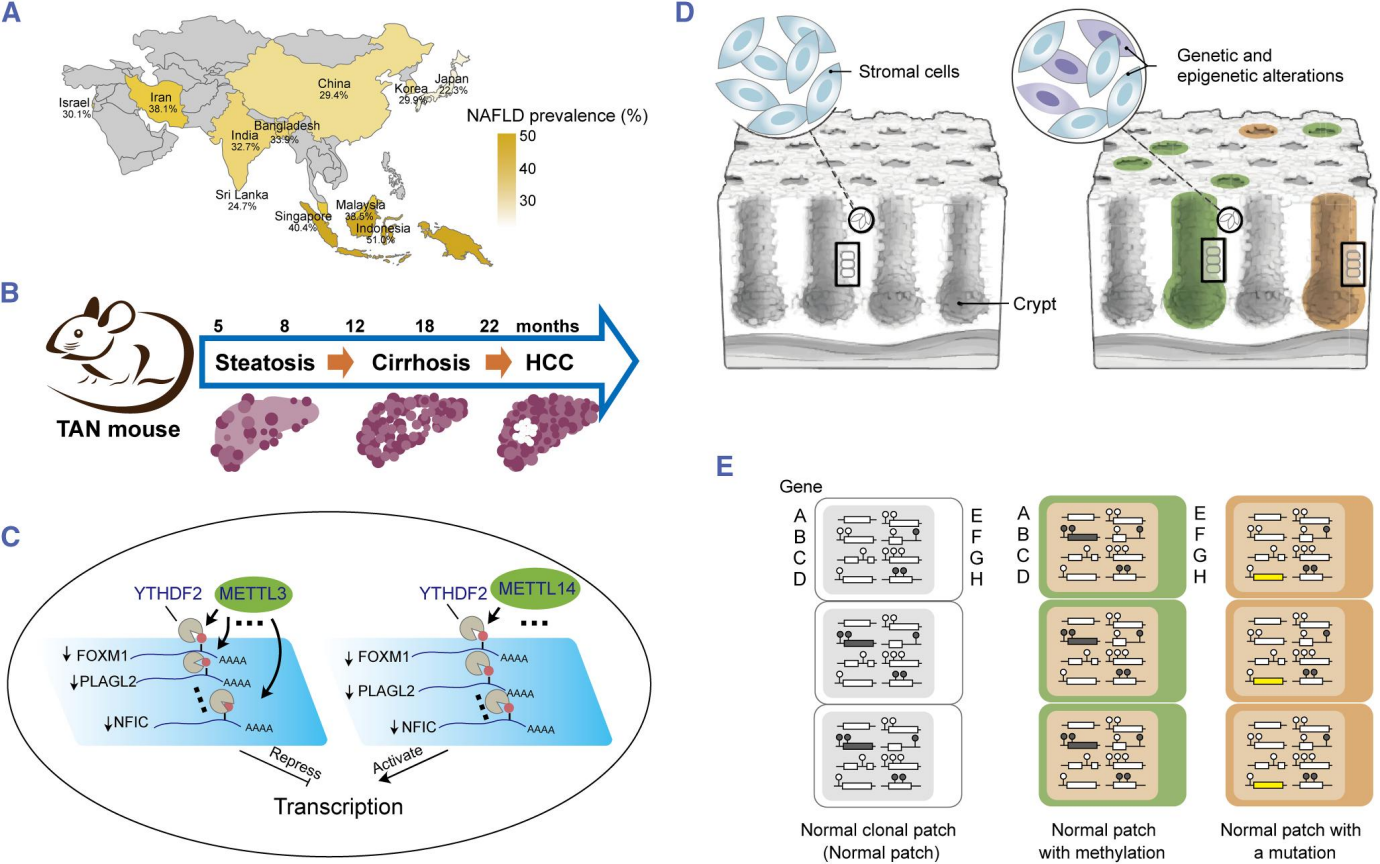
The progress of m6A epitranscriptome in the establishment of HCC and NAFLD. (A) Rising incidence of NAFLD and HCC. Reproduced with permission.^5^ Copyright 2022, Elsevier. (B) NAFLD and HCC development are strongly correlated with epigenetic changes. Reproduced with permission.^6^ Copyright 2022, Elsevier. (C) The probable consequences of m6A alteration in NAFLD and HCC. Reproduced with permission.^11^ Copyright 2021, Oxford University Press. (D–E) Major breakthroughs in RNA‐modification‐mediated gene expression regulation. Reproduced with permission.^7^ Copyright 2021, The Authors, published by American Association for the Advancement of Science. HCC, hepatocellular carcinoma; NAFLD, non‐alcoholic fatty liver disease.

Here, we shall provide the most current discoveries on mapping m6A epitranscriptome in the molecular pathways participating in the development of NAFLD and HCC, with special attention to the impacts of m6A modification on the progression of HCC and elaborate on its possible roles in the progression of NAFLD to HCC, which is illustrated in Figure 2. Finally, we will discuss the probable consequences of m6A alteration in NAFLD and HCC diagnosis and treatment.

## 2 RNA m6A MODIFICATION

The m6A alteration is an RNA modification that has been preserved throughout evolution and may be found in a wide range of animals, from bacteria to humans.^15^ In addition, Chemical modification at m6A is shown to be the most common of all modifications to mRNA and lncRNA.^10^ On average, 0.1%–0.4% of mRNA adenosines are changed by m6A, and each transcript has two to three such sites.^16^ Researchers have found that the m6A alteration is reversible and may be dynamically controlled by three identical components known as “writers,” “erasers,” and “readers,” implying that these proteins have the capacity to modulate biological processes.^17^ Specifically, components that promote the synthesis of m6A methylation are called “writers,” whereas demethylating components, or “erasers,” play a crucial part in m6A modification^18^; a collection of molecules known as readers can decode m6A methylation and provide functional signals.^19^

The balance of the writer and eraser proteins in m6A controls the reversible nature of the installation process. Writer proteins are methyltransferase complexes that facilitate the deposit of m6A in mRNA, such as METTL3.^20^, ^21^, ^22^, ^23^, ^24^ Eraser proteins catalyze m6A removal from mRNA transcripts, such as FTO and ALKBH5.^25^, ^26^, ^27^ Since writer and eraser proteins are present, the m6A modification process is a dynamic and reversible mechanism that may rapidly fine‐tune the fate of mRNA transcripts, enabling quick adjustment to sudden environmental changes like hypoxia and damage. Reader proteins, which may be categorized into three types, are m6A binding proteins that identify m6A sites in the cytoplasm. YTH (YT521‐B homology) domains are evolutionarily conserved and found in class I m6A reader proteins, including YTHDF1‐3 and YTHDC1‐2.^28^, ^29^, ^30^, ^31^, ^32^ YTHDF1 and YTHDF3 recruitment to m6A locations improves transcripts from messenger RNA, while YTHDC1 specifically interacts with m6A marks and regulates the alternate splicing of mRNA.^33^ RNA transcripts with m6A alterations are targeted for destruction by YTHDF2.^34^ There are three heterogeneous nuclear ribonucleoproteins (hnRNPs) in the class II m6A reader proteins: hnRNPC, hnRNPG, and hnRNPA2B1.^35^ Researchers observed hnRNPA2B1 binding to m6A sites increases initial miRNA processing.^36^ IGF2BP1‐3, members of the IGFBP family, are class III m6A reader proteins.^37^ The promotion of mRNA stability and translation by the binding of IGF2BP1‐3 to mRNA that has been modified with m6A.^37^

**FIGURE 3:**
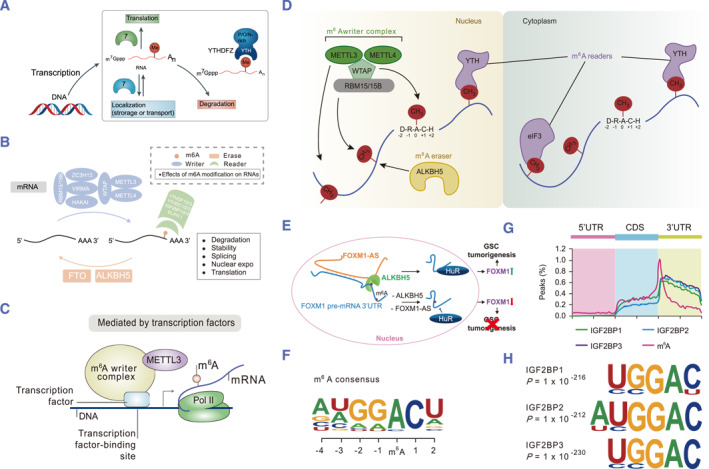
The most frequent chemical change seen in eukaryotic mRNA and lncRNA is the M6A modification. (A) M6A modification is a universally present RNA alteration that has seen little change throughout evolution. Reproduced with permission.^15^ Copyright 2014, Springer Nature. (B) M6A modification are composed of writers, erasers, and readers. Reproduced under terms of the CC‐BY license.^18^ Copyright 2022, The Authors, published by Springer Nature. (C) A collection of molecules known as readers can decode m6A methylation and provide functional signals. Reproduced with permission.^19^ Copyright 2019, Springer Nature. (D) YTHDC1 preferentially binds to m6A, enhancing mRNA translation. Reproduced with permission.^33^ Copyright 2018, Elsevier. (E) Most mRNA processing characteristics are strongly regulated by the M6A alteration. Reproduced with permission.^39^ Copyright 2017, Elsevier. (F–H) The main miRNA processing is facilitated and the movement of mRNAs through the nucleocytoplasm is mediated by the binding of hnRNPA2B1 to m6A sites. Reproduced with permission.^37^ Copyright 2018, Springer Nature.

In conclusion, m6A modification strictly regulates the majority of mRNA processing functions, such as translation, mRNA stability, and pre‐mRNA splicing, and plays a significant part in development,^38^ cell growth,^39^ and tumorigenesis,^40^ which is illustrated in Figure 3.

## 3 ROLES OF RNA m6A METHYLATION IN NAFLD

**FIGURE 4:**
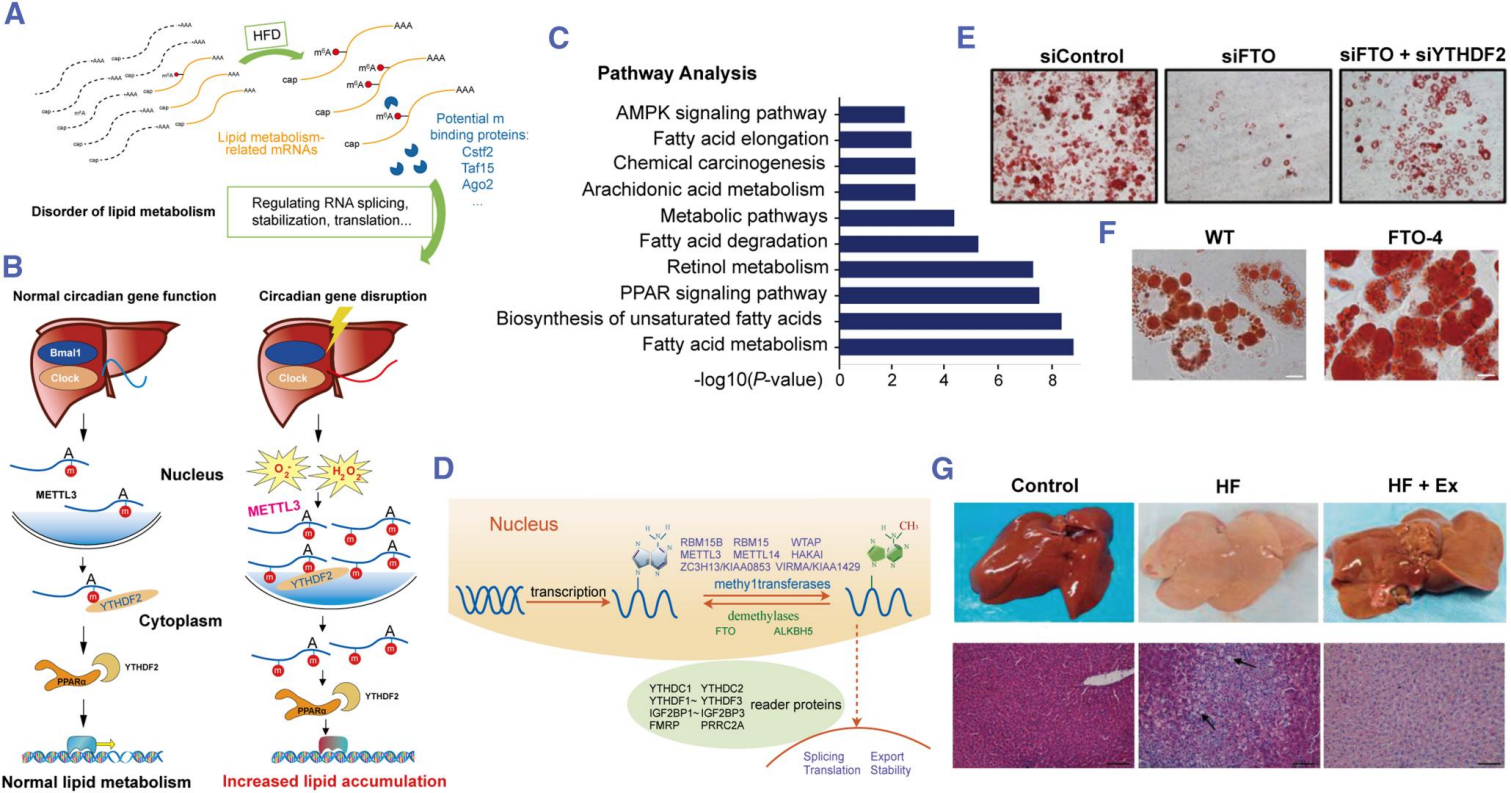
M6A methylation is crucial in the development of NAFLD. (A) The emergence of NAFLD is strongly correlated with M6A change. Reproduced with permission.^45^ Copyright 2019, Future Medicine, Ltd. (B) Knockdown of METTL3, an m6A methyltransferase, could lead to the lipid accumulation in liver. Reproduced with permission.^47^ Copyright 2018, The Authors, published by Elsevier. (C) Significantly, higher levels of m6A enrichment and mRNA expression of lipogenic genes are seen in the NAFLD model. Reproduced with permission.^46^ Copyright 2020, John Wiley and Sons. (D) The hepatic tissue of NAFLD has an increased amount of FTO. Reproduced under terms of the CC‐BY license.^50^ Copyright 2020, The Authors, published by Springer Nature. (E) Through methylating m6A, YTHDF2 can counteract FTO‐mediated adipogenesis. Reproduced with permission.^54^ Copyright 2018, Elsevier. (F) FTO is shown to regulate gluconeogenesis in the liver. Reproduced under terms of the CC‐BY license.^49^ Copyright 2015, The Authors, published by Springer Nature. (G) By inhibiting FTO, activation of the PI3K/AKT signaling pathway may enhance the progress of NAFLD. Reproduced under terms of the CC‐BY license.^51^ Copyright 2018, The Authors, published by Associacao Brasileira de Divulgacao Cientifica. NAFLD, non‐alcoholic fatty liver disease; PI3K, phosphatidylinositol 3‐kinase.

NAFLD has become the most prevalent chronic liver disease globally, due to its high incidence and propensity for progression to other liver disorders, such as NASH, liver fibrosis, cirrhosis, and HCC.^41^ De novo lipogenesis (DNL), or the intake of fatty acids, is more than compensated by fatty acid oxidation or very low density lipoprotein, which is thought to be the cause of NAFLD.^42^ NAFLD is defined by hepatic steatosis in the absence of a history of significant alcohol intake or any identified liver diseases,^43^ and hepatic steatosis is caused by metabolic abnormalities in DNL, fatty acid absorption, fatty acid oxidation, and triglyceride export.^44^ m6A methylation plays a critical function in the control of obesity and type 2 diabetes, which are the main causes of NAFLD. According to research, m6A mutations are strongly linked to the development of NAFLD.^45^ Researchers discovered that m6A enrichment and mRNA expression of lipogenic genes are dramatically elevated in leptin receptor‐deficient db/db mice using m6A‐sequencing and RNA‐sequencing, which is a rodent model of NAFLD.^46^ Overall, the functions were summarized below and illustrated in Figure 4.

### 3.1 Writers

A recent research found that knocking down METTL3, a m6A methyltransferase, decreased hepatic m6A abundance on peroxisome proliferators‐activated receptors alpha (PPAR) but increased PPAR mRNA lifespan, resulting in increased PPAR expression and decreased fat buildup in the liver.^47^

### 3.2 Erasers

FTO, a m6A demethylase, has been demonstrated in mice to control gluconeogenesis and thermogenesis in adipose tissues,^26^, ^48^, ^49^ and m6A demethylation favorably influenced adipogenesis.^26^ FTO (R316A) mutant lacks demethylation function and is unable to modulate mitochondrial and TG levels, the level of FTO is enhanced in the hepatic tissue of NAFLD patients who are hyperglycemic and hyperinsulinemic. This can up‐regulate TG deposition while down‐regulating mitochondrial content,^50^ indicating that FTO can modify m6A levels in hepatocytes, which can impact mitochondrial content and fat metabolism.^13^ By preventing FTO‐mediated hepatocyte regeneration, the phosphatidylinositol 3‐kinase/AKT signaling pathway may contribute to the development of NAFLD.^51^ Increased FTO levels can up‐regulate intracellular TG levels, which in turn encourages the production of hepatic fat. These lipogenic genes include FASN, stearoyl‐CoA desaturase (SCD), and MOGAT1.^13^ Analogously, in vitro and vivo research of glucocorticoid‐induced NAFLD demonstrate that glucocorticoid receptor‐dependent FTO transactivation and m6A demethylation on the mRNA of lipogenic genes, such as SREBF1 and SCD, activation of these lipogenic genes and lipid accumulation in the liver.^52^

### 3.3 Readers

Researchers have found that includes two in the YT521‐B homology domain (YTHDC2), A m6A reader may detect a particular m6A methylation alteration on the transcripts of target genes and pass them along for further processing, which modifies the levels of those genes' mRNA.^53^ In NAFLD livers, YTHDC2, which binds to stearoyl‐CoA desaturase 1 (SCD1), FASN, SREBP‐1c, ACC1, and reduces the stability of their mRNA and blocks gene expression, is markedly downregulated, leading to the accumulation of TGs and the progression of NAFLD.^46^ Through m6A methylating, YTHDF2 can also counteract FTO‐mediated adipogenesis.^54^

## 4 ROLES OF RNA m6A METHYLATION IN HCC

**FIGURE 5:**
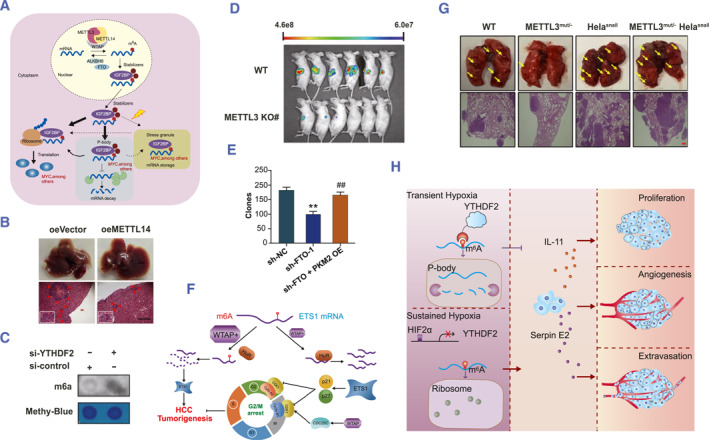
M6A methylation is a vital regulator of HCC development. (A) The mechanism of m6A modification in HCC. Reproduced with permission.^37^ Copyright 2018, The Authors, published by Springer Nature. (B) METTL14 inhibits HCC metastatic potential by altering a m6A‐dependent mechanism. Reproduced with permission.^59^ Copyright 2017, John Wiley and Sons. (C) Knockdown of YTHDF2 suppresses the proliferation in vivo. Reproduced with permission.^62^ Copyright 2017, The American Society for Biochemistry and Molecular Biology, Inc. (D) METTL3 is frequently over‐expressed in human HCC and drives the development of HCC cells. Reproduced with permission.^55^ Copyright 2018, John Wiley and Sons. (E) YTHDF2 enhanced the mRNA degradation of HCC cells by identifying mRNA m6A sites **shFTO‐1 versus sh‐NC, ^##^shFTO‐1 + PKM2 versus sh‐NC. Reproduced with permission.^60^ Copyright 2019, E‐Century Publishing Corporation. (F) The M6A writer complex is overexpressed in HCC. Reproduced under terms of the CC‐BY license.^57^ Copyright 2019, The Authors, published by Springer Nature. (G) The whole mRNA m6A level rose considerably during HCC Epithelial mesenchymal transition. Reproduced under terms of the CC‐BY license.^56^ Copyright 2019, The Authors, published by Springer Nature. (H) YTHDF2 alters the microenvironment of HCC by inducing inflammation and vascular remodeling. Reproduced under terms of the CC‐BY license.^63^ Copyright 2019, The Authors, published by Springer Nature. HCC, hepatocellular carcinoma.

Recently, the significance of m6A alteration in HCC has come to light more and more. Human HCC has a higher amount of global m6A modification.^37^ Overall, the functions were summarized below and illustrated in Figure 5.

### 4.1 Writers

Recently, researchers have found that HCC is connected to m6A authors like METTL3 and METTL14. By thoroughly examining writers and erases in various public cohorts. According to Chen et al., METTL3 is frequently up‐regulated in human HCC and encourages colony formation, growth, and migration of HCC cells in culture as well as tumorigenicity, growth, and lung metastasis of HCC in vivo by suppressing SOCS2 mRNA, whose degradation depends on the m6A reader protein‐dependent pathway.^55^ Mechanistically, METTL3 enhances the degradation of tumor suppressor gene SOCS2 mRNA by promoting m6A modification on the 3′ end of SOCS2 mRNA in a YTHDF2‐dependent manner.^55^ Additionally, patients with HCC who have an overexpression of METTL3 have a worse prognosis.^55^ Researchers discovered that the global mRNA level of m6A rose considerably via the Epithelial mesenchymal transition process, and they pinpointed Snail as a METTL3‐mediated m6A alteration target, which cooperates with YTHDF1 to encourage Snail's protein translation and contribute to HCC metastasis.^56^ The m6A writer complex also includes WTAP and KIAA1429, which are elevated in HCC and associated with a poor prognosis.^57^, ^58^ By regulating the m6A‐dependent primary MicroRNA 126 process by the microprocessor protein DGCR8, METTL14 inhibits the metastatic potential of HCC. As a result, down‐regulation of METTL14 plays a vital role in HCC metastasis and is viewed as a poor prognostic indicator for recurrence‐free survival of HCC.^59^

### 4.2 Erasers

FTO levels are increased in HCC tissue and cells. FTO overexpression is correlated to a poor prognosis in HCC, and FTO knockdown decreases proliferation and in vivo tumor development while inducing G0/G1 phase arrest. Mechanically, FTO promotes HCC growth by stimulating PKM2 mRNA demethylation and facilitating protein translation.^60^

### 4.3 Readers

Several m6A readers, as well as writers and erasers, have been implicated in HCC. Through increasing HCC cell cycle progression and metabolism, the m6A reader YTHDF1 is highly increased in HCC and is positively linked with the disease stage.^61^ The expression of YTHDF2 in HCC remains debatable. It has been discovered YTHDF2 increased mRNA degradation in HCC cells by finding mRNA m6A sites, hence increasing HCC growth.^62^ The 3′ UTR of the mRNA for YTHDF2 interacts to miR‐145 in this instance, severely inhibiting its expression. YTHDF2 is probably increased in the HCC cohort since miR‐145 is commonly downregulated in HCC and negatively correlates with YTHDF2 expression.^62^ On the other hand, Hou et al. found expression of YTHDF2 was decreased in HCC, which was linked with various aggressive clinical characteristics, and that YTHDF2 loss disturbed m6A‐dependent mRNA degradation of IL11 and SERPINE2 mRNA. IL11 and SERPINE2 overexpression alters the HCC microenvironment by increasing the tumor environment.^63^

## 5 CONCLUSIONS AND PERSPECTIVES

NAFLD and HCC, as the most common and severe form of liver diseases respectively, have a correlation in the molecular mechanisms of pathogenesis. Methylation of RNA m6A has several functions in the control of hepatic growth, adipogenesis, as well as the progression of NAFLD and HCC. Both METTL3 and FTO play important roles in NAFLD and HCC, implying that they may be the bridge connecting these two diseases. Of note, the possibility of other m6A proteins participating in the progression of NAFLD to HCC cannot be ruled out.

M6A methylation is a modification found in RNA molecules that plays an important role in regulating gene expression and cell fate determination. Firstly, for certain types of tumors, M6A methylation is considered as a targetable biomarker and may become a target for therapeutic strategies. For instance, ALKBH5 has a cancer suppressor effect, which can reduce the expression of LYPD1 in HCC cells in an M6a‐dependent manner, thus becoming a potential target for HCC.^64^ Secondly, by altering the status of M6A methylation, such as using M6A methyltransferase inhibitors or demethylating agents, gene expression at the RNA level can be regulated, which may achieve the effect of precision medicine treatment. In addition, combining M6A methylation with other diagnostic techniques, such as genome sequencing, proteomics, and metabolomics, can also improve the accuracy of clinical diagnosis and prediction. In summary, M6A methylation has a wide range of clinical applications, especially in the treatment of NAFLD and HCC.

In conclusion, methylation m6A levels may be a viable marker for early detection of NAFLD and HCC, and m6A may be a promising target for the therapy of NAFLD‐derived HCC. The functions of m6A methylation genes were concluded in Table 1. However, recent research only paints a partial picture of liver diseases, and further studies on RNA m6A modification may assist us to comprehend their functions in hepatic diseases.

**TABLE 1:** The functions of RNA m6A methylation genes in both NAFLD and HCC.

Types	Genes	Functions in NAFLD/HCC	PMID
Writer	METTL3	Knocking down METTL3 decreased fat buildup in the liver	30428350
	METTL5	METTL5 could stabilize c‐Myc to reprogram glucose metabolism in HCC	36602428
	METTL14	METTL14 inhibits the metastatic potential of HCC	27774652
	METTL16	Deletion of METTL16 in HCC lines decreases the abundance of m6A‐modified mRNA and inhibits the translational level of HCC cells	35145225
	WTAP	WTAP facilitates the progression of HCC via silencing of ETS1	31438961
Eraser	ALKBH3	ALKBH3 could promote HCC cell proliferation and invasion	36098205
	ALKBH5	ALKBH5 regulates PD‐L1+ macrophage infiltration and promotes HCC progression	35982895
	FTO	FTO could control gluconeogenesis and thermogenesis in adipose tissues of NAFLD	25412662
Reader	YTHDC2	YTHDC2 accumulates TGs and counteracts FTO‐mediated adipogenesis in NAFLD	32150756
			30305247
	YTHDF1	YTHDF1 drives hypoxia‐induced autophagy and malignancy of HCC by promoting ATG2A and ATG14 translation	33619246
	YTHDF2	YTHDF2 promotes the HCC stem cell phenotype and cancer metastasis by regulating OCT4	32366907
	YTHDF3	YTHDF3‐mediated m6A modification could regulate the glycolysis of HCC	36471428

Abbreviations: HCC, hepatocellular carcinoma; NAFLD, non‐alcoholic fatty liver disease.

## AUTHOR CONTRIBUTIONS

Jinglin Wang and Haozhen Ren conceived and designed the experiments. Yuyan Chen, Lu Zhang, and Zhengyi Zhu searched the related literature. Yuyan Chen drafted the manuscript. All authors read and approved the final manuscript.

## CONFLICT OF INTEREST STATEMENT

All authors declare that there are no competing interests.
